# Electrocupuncture combined rehabilitation therapy for upper limb spasticity after stroke: A protocol for systematic review and meta-analysis

**DOI:** 10.1097/MD.0000000000027963

**Published:** 2021-11-24

**Authors:** Huijuan Lou, Zhanxin Li, Tingting Pang, Xinxin Zhang, Meng Meng, Kang Yang, Hongshi Zhang, Yufeng Wang, Deyu Cong

**Affiliations:** a Department of Acupuncture and Tuina, Changchun University of Chinese Medicine, Changchun, China; b Department of Nursing, Changchun University of Chinese Medicine, Changchun, China; c Department of Tuina, Traditional Chinese Medicine Hospital of Jilin Province, Changchun, China.

**Keywords:** electrocupuncture, meta-analysis, rehabilitation, stroke, systematic review, upper limb spasticity

## Abstract

**Background:**

The purpose of this study was to evaluate the effectiveness and safety of electroacupuncture combined with rehabilitation in the treatment of spasticity after stroke.

**Methods:**

To collect relevant literature, we will research following databases: Medicine, PubMed, Embase, Web of Science, Cochrane Library, China National Knowledge Infrastructure, Wan-Fang Database, Chongqing VIP Chinese Science and Technology Periodicals Database and China Biomedical Database; the time is from its creation to May 2021, and the language is limited to Chinese and English. In addition, we will retrieve other literature resources, including the Chinese Clinical Trial Register and conference papers. Two reviewers will independently complete the literature screen and data extraction, and quality assessment of the included studies will be independently completed by two other researchers. The primary outcomes included the Modified Ashworth scale (MAS) and the simplified Fugl-Meyer Assessment scale (SFMA). The Modified Barthel Index (MBI), the China Stroke Scale (CSS), and adverse reactions as secondary outcomes were assessed. RevMan V.5.4.1 software will be used for meta-analysis, and the Grading of Recommendations Assessment, Development, and Evaluation (GRADE) will be used to assess the quality of evidence.

**Results:**

This systematic review will provide a high-quality synthesis to evaluate the efficacy and safety of electroacupuncture combined with rehabilitation therapy in the treatment of upper limb spasticity after stroke, providing a reference for the safe and effective treatment of upper limb spasm after stroke.

**Conclusion:**

This study provides evidence that electroacupuncture combined with rehabilitation therapy is effective.

**Ethics and dissemination:**

The protocol of the systematic review does not require ethical approval because it does not involve humans. This article will be published in peer-reviewed journals and presented at relevant conferences.

**Systematic review registration:**

INPLASY202160005.

## 1 Introduction

Stroke is the first and third most disabling disease in China and the third in the world. This disease has high morbidity, mortality, and disability rates, and is a serious threat to human health.^[1–4]^ Limb spasm is the main complication of stroke.^[5]^ Data show that 90% of patients will have limb spasm within 3 weeks after the onset of stroke, and more than half of the patients with upper limb spasm are the main performance.^[6,7]^ Limb spasticity is a type of abnormal movement pattern, which is mainly caused by the loss of control of lower motor neurons after damage to the upper motor nerve center, resulting in hyperreflexia of the spinal cord retch.^[7,8]^ Spasticity of the upper limb develops from flaccid paralysis and is characterized by increased muscle tension, upper limb flexion, and finger flexion.^[9]^ The upper limb is an important carrier for us to complete information communication and emotional communication, which undertakes most of our daily activities. Therefore, if the upper limb is in spasm and cannot be recovered, it will have a great impact on the quality of life and psychological state of post-stroke patients.^[10,11]^

These treatments are recommended by the American Heart Association (AHA)/American Stroke Association (ASA) adult stroke treatment rehabilitation guidelines, including oral drugs, botulinum toxin injections, and normal limb position placement.^[12]^ These interventions can help improve the symptoms of spasms and hemiplegia to some extent. However, the effect of recovery varies from person to person, and some interventions, such as oral drugs and botulinum toxin injection, have different side effects.^[13,14]^ In recent years, an increasing number of clinical studies have adopted integrated therapy to intervene in diseases where the use of electroacupuncture combined with modern rehabilitation therapy is a popular option. While many clinical studies have reported its positive impact on upper limb spasms after stroke, there is no scientific evidence. Therefore, the purpose of this systematic review was to assess the effectiveness and safety of electroacupuncture combined with rehabilitation for limb spasm after stroke and to provide better clinical decision-making.

## 2 Methods and analysis

The study was conducted following the guidelines of the Preferred Reporting Items for Systematic Review and Meta-analysis Protocol (PRISMA-P).^[15]^ The study protocols were funded using a protocol registry. This systematic review protocol was registered on the INPLASY website. Registration: INPLASY202160005.

### 2.1 Inclusion criteria

#### 2.1.1 Types of participants

All patients should be diagnosed with stroke and show symptoms of upper limb muscle spasm, and should be older than 18 years of age. However, race, gender, and educational status are not limited. The diagnosis of stroke should meet WHO criteria.^[16]^ Participants with unstable vital signs or inability to cooperate with rehabilitation treatment should be excluded, such as patients with impaired hearing, visual and cognitive or severe infection, organ dysfunction, and so on.

#### 2.1.2 Types of interventions

The intervention in the experimental group should be electroacupuncture combined with rehabilitation therapy, and the interventions of the control group should only be rehabilitation therapy. The methods of electroacupuncture include Filifrom-Needle electrocupuncture, Fire-Needle electrocupuncture, Scalp electrocupuncture Abdominal electrocupuncture, and Electroelectrocupuncture; the methods of rehabilitation training are not limited (including all types of rehabilitation training methods for upper limb spasticity after stroke, such as Bobath Technology, Rood Technology, Brunnstrom Therapy, Excercise Relearning Therapy, and Proprioceptive Neuromuscular Facilitation). If there are other adjuvant therapies, the two groups should be consistent.

#### 2.1.3 Types of studies

1. Inclusion: We will include only randomized controlled clinical trials (RCTs) of electroacupuncture combined with rehabilitation therapy for upper limb spasticity after stroke.
2. Exclusion: We will exclude any other literature including non-randomized clinical controlled trials, retrospective research literature, conference abstracts, case reports, repeated published literature, and literature of information without data.

#### 2.1.4 Types of outcomes

##### 2.1.4.1 Main outcomes

We will include the Modified Ashworth Scale (MAS) and Simplified Fugl-Meyer Assessment scale (SFMA) as the main outcomes. The MAS will be used to evaluate the muscle tone of the patient's upper limbs and divided into five grades according to severity. The SFMA, 100 points in total, can assess movement function of patient's limbs (including upper and lower limbs), yet only the part of SFMA about the upper limbs was used (66 points) in this study.

##### 2.1.4.2 Secondary outcomes

1. Modified Barthel Index (MBI) used to evaluate the daily living ability of patients with stroke.
2. China Stroke Scale (CSS) used to assess the neurological deficit of stroke patients.
3. adverse reactions.

### 2.2 Data sources and search methods

#### 2.2.1 Electronic searches

We will collect relevant articles by searching the following databases: PubMed, Web of Science, Medicine, EMBASE, Cochrane Library, China National Knowledge Infrastructure, China Biomedical Literature Database, China Science Journal Database, and Wan-Fang Database. All databases will be searched from creating to May 1, 2021, by the following words: Stroke, Post-stroke, Spastic paralysis, Spastic hemiplegia, Upper limb spasticity, Upper spastic paraparesis, Upper muscle paralysis, electrocupuncture, Acupoint, Rehabilitation, Habilitation, RCT, etc. The research strategy for PubMed is presented in Table 1.

**Table 1:** Search strategy used in PubMed.

No	Search items
#1	Spastic paralysis (All Fields)
#2	Spastic hemiplegia (All Fields)
#3	Spastic paraparesis (All Fields)
#4	Upper motor nearon paralysis (All Fields)
#5	Central paralysis (All Fields)
#6	Muscle spasticity (All Fields)
#7	Stiff paralysis (All Fields)
#8	Upper limb spasticity (All Fields)
#9	Upper limb spasmodic hemiplegia (All Fields)
#10	#1 OR #2-#9
#11	Stroke (All Fields)
#12	Post-stroke (All Fields)
#13	Apoplexy (All Fields)
#14	Cerebrovascular disorder (All Fields)
#15	Brain ischemia (All Fields)
#16	Intracranial arterial disease (All Fields)
#17	Intracranial embolism and thrombosis (All Fields)
#18	Intracranial haemorrhages (All Fields)
#19	#11 OR #12-#18
#20	Electrocupuncture (All Fields)
#21	Acupoint (All Fields)
#22	Meridians (All Fields)
#23	Electrocupuncture therapy (All Fields)
#24	Rehabilitation (All Fields)
#25	Habilitation (All Fields)
#26	#20 OR #21-#25
#27	Randomized controlled trial (All Fields)
#28	Controlled clinical trial (All Fields)
#29	Randomized (All Fields)
#30	Randomly (All Fields)
#31	#27 OR #28-#30
#32	#10 AND #19 AND #26 AND #31

#### 2.2.2 Searching for other resources

We will search the reference list of the included studies and existing systematic reviews related to our topic. We will also search for other literature resources, including the Chinese Clinical Trial Register, conference papers, and other related gay literature to make our search as complete as possible.

### 2.3 Data collection and export

**Figure 1:**
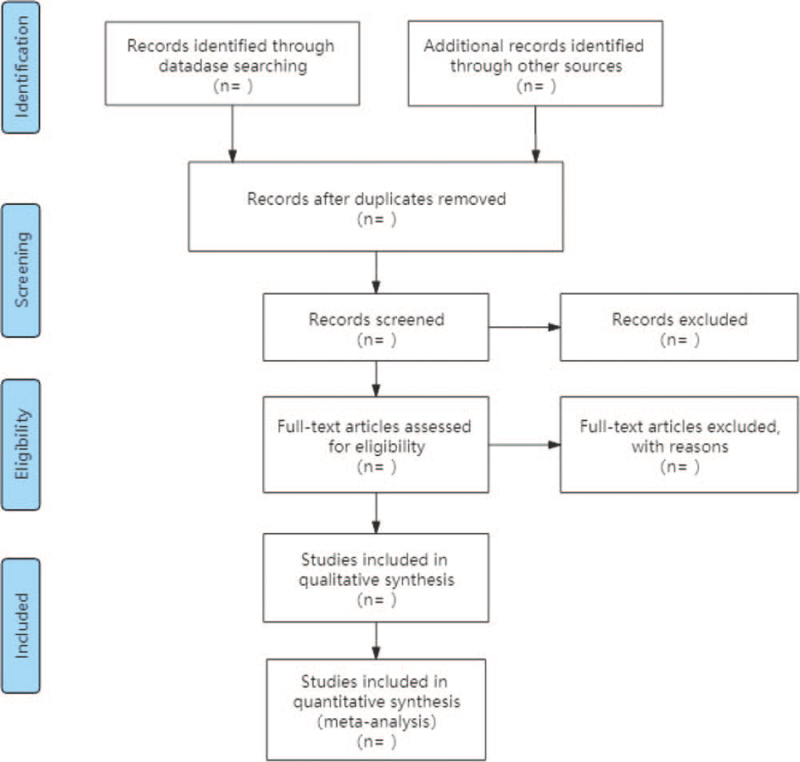
Flow diagram of study selection process.

Two researchers independently screened the literature according to eligibility criteria. First, duplicate articles were eliminated using EndNote V.x 9.0, and excluded articles that did not meet the inclusion criteria by reading the title and subject. Second, they will perform a screen again of the remaining articles by reading the full text according to the inclusion and exclusion criteria and determine whether it is available for the systematic review. We will also record the excluded papers and explain the reasons for this; the specific screening process is shown in Figure 1. If there is disagreement during, the third researcher will be invited to make a decision.

### 2.4 Data extraction and analysis

Data extraction will be performed independently by two reviewers (who and who), and the results will be cross-matched. When the differences and opinions are inconsistent, they should be settled through discussion. If the differences encountered cannot be resolved through discussion, a third researcher will be invited to resolve them. Excel will be used to extract data, including the first author, country, year of publication, patient characteristics, number of participants, interventions, outcomes, results, main conclusions, conflicts of interest, ethical approval, and other information. If necessary, we will contact the corresponding author by e-mail to obtain more accurate data.

### 2.5 Assessment of risk of bias in the included studies

Two researchers will independently evaluate the bias risk, including studies using the assessment tool of risk bias in the Cochrane Handbook V.5.1.0. The contents included random sequence generation, allocation sequence concealment, blinding of participants and personnel, outcome assessors, incomplete outcome data, selective outcome reporting, and other sources of bias. The assessment results were rated as low-risk, high-risk, or uncertain risk. In the process, if there is disagreement, a third reviewer will be invited to make a decision.

### 2.6 Assessment of heterogeneity

The heterogeneity test will be carried out among all studies included using the *I*^2^ statistic. No significant heterogeneity was observed when *I*^2^ was <50%. Otherwise, if the result of *I*^2^ was more than 50%, we believe that there was obvious heterogeneity, and subgroup analysis and sensitivity analysis were conducted to investigate the sources of heterogeneity.

### 2.7 Assessment of reporting biases

We will analyze the quality of publication bias using RevMan 5.4.1 software in inverted funnel plots and performing Egger's test when there were >10 trials included in the meta-analysis.

### 2.8 Data synthesis

The meta-analysis of data from included outcomes will be performed using the RevMan V.5.4.1, and we will choose a randomized or fixed effect model for data statistics according to the results of the heterogeneity test. The enumeration data were expressed as relative risk (RR), and the weight mean difference (WMD) was used as the measurement data; each effect amount was expressed in 95% confidence interval (CI). The specific methods were as follows: If the heterogeneity was low (*I*^2^ < 50%, the fixed-effects model was used for data synthesis. If there is high heterogeneity (*I*^2^ > 50%), the random-effects model will be used for data synthesis after excluding possible heterogeneity sources. The investigation methods included subgroup and sensitivity analyses. If data cannot be synthesized, we provide a descriptive analysis to solve this problem.

### 2.9 Subgroup analysis

If there was high heterogeneity (*I*^2^ > 50%) among the included studies, we conducted a subgroup analysis to analyze the sources of heterogeneity according to the following factors: age, sex, race, course, sample size, different methods of electroacupuncture or rehabilitation, and other possible factors affecting the results.

### 2.10 Sensitivity analysis

To test the stability and reliability of the results of this study, we conducted a sensitivity analysis according to the following points: method quality, sample size, and missing data. After that, we will perform a data analysis again and compare the results. If there was no directional change after the sensitivity analysis, the results were stable.

### 2.11 Grading the quality of evidence

We will use the Grading of Recommendations Assessment, Development, and Evaluation to assess confidence in cumulative evidence.^[17]^ The risk of publication, heterogeneity, indirectness, imprecision, and publication bias were assessed, and the results were divided into four levels: high, moderate, low, and very low.

### 2.12 Ethics and dissemination

Ethical approval was not required because no primary information of individual patients was collected. We will publish this article in a peer-reviewed journal.

## 3 Discussion

Stroke, a common clinical emergency, can easily lead to death, and most surviving patients have varying degrees of functional impairment. Among them, limb spasm has attracted wide attention as a major problem in rehabilitation after stroke.^[18]^ At present, the mainstream treatment method is rehabilitation training, but there are long treatment cycles, easy to leave sequelae, and other defects. Electroacupuncture, as a non-drug therapy in traditional Chinese medicine, has been widely used in clinical practice, and research results have shown that electroacupuncture has a good effect on limb spasticity after stroke. Its mechanism of action may be to stimulate specific neurotransmitters to balance the tension of antagonistic and spasm muscles and promote the recovery of limb motor function.^[19,20]^ The combination of traditional electroacupuncture and modern rehabilitation techniques can greatly shorten the clinical treatment cycle and improve treatment effectiveness. However, due to the lack of structured methods, effective evidence is still needed to support this conclusion. This study will conduct a systematic review and meta-analysis of data from relevant randomized controlled trials to verify its efficacy and safety and provide evidence-based medicine evidence for the clinical treatment of this disease.

## Author contributions

Deyu Cong contributed to the conception of the study. Huijuan Lou and Zhanxin Li drafted and revised the manuscript. The search strategy was developed by all the authors and will be performed by Xinxin Zhang and Kang Yang, TingTing Pang and Meng Meng will independently screen the potential studies and extract data from the included studies. Assess the risk of bias and complete Hongshi Zhang and Zhanxin Li. Huijuan Lou will complete data synthesis. Yufeng Wang arbitrate disagreement Deyu Cong ensured the absence of any errors. All authors approved the publication of the protocol.

**Data curation:** Zhanxin Li.

**Formal analysis:** Tingting Pang.

**Investigation:** Meng Meng, Hongshi Zhang.

**Methodology:** Xinxin Zhang, Kang Yang.

**Validation:** Yufeng Wang.

**Writing – original draft:** Huijuan Lou.

**Writing – review & editing:** Deyu Cong.
